# Loggerhead sea turtle (*Caretta caretta*) diving changes with productivity, behavioral mode, and sea surface temperature

**DOI:** 10.1371/journal.pone.0220372

**Published:** 2019-08-07

**Authors:** Autumn R. Iverson, Ikuko Fujisaki, Margaret M. Lamont, Kristen M. Hart

**Affiliations:** 1 Cherokee Nation Technologies, contracted to Wetland and Aquatic Research Center, United States Geological Survey, Davie, Florida, United States of America; 2 Fort Lauderdale Research and Education Center, University of Florida, Davie, Florida, United States of America; 3 Wetland and Aquatic Research Center, United States Geological Survey, Gainesville, Florida, United States of America; 4 Wetland and Aquatic Research Center, United States Geological Survey, Davie, Florida, United States of America; Deakin University, AUSTRALIA

## Abstract

The relationship between dive behavior and oceanographic conditions is not well understood for marine predators, especially sea turtles. We tagged loggerhead turtles (*Caretta caretta*) with satellite-linked depth loggers in the Gulf of Mexico, where there is a minimal amount of dive data for this species. We tested for associations between four measurements of dive behavior (total daily dive frequency, frequency of dives to the bottom, frequency of long dives and time-at-depth) and both oceanographic conditions (sea surface temperature [SST], net primary productivity [NPP]) and behavioral mode (inter-nesting, migration, or foraging). From 2011–2013 we obtained 26 tracks from 25 adult female loggerheads tagged after nesting in the Gulf of Mexico. All turtles remained in the Gulf of Mexico and spent about 10% of their time at the surface (10% during inter-nesting, 14% during migration, 9% during foraging). Mean total dive frequency was 41.9 times per day. Most dives were ≤ 25 m and between 30–40 min. During inter-nesting and foraging, turtles dived to the bottom 95% of days. SST was an important explanatory variable for all dive patterns; higher SST was associated with more dives per day, more long dives and more dives to the seafloor. Increases in NPP were associated with more long dives and more dives to the bottom, while lower NPP resulted in an increased frequency of overall diving. Longer dives occurred more frequently during migration and a higher proportion of dives reached the seafloor during foraging when SST and NPP were higher. Our study stresses the importance of the interplay between SST and foraging resources for influencing dive behavior.

## Introduction

How an animal moves across spatial and temporal scales is fundamental to its ecology. These movements can influence its ability to survive and have impacts on population and ecosystem dynamics and ultimately evolution [1]. Determining factors that drive animal movement behavior is an important part of movement ecology frameworks [1]. Sea turtle diving is a particularly interesting movement behavior as they demonstrate the longest reported breath-hold dives of all marine animals [2] and typically spend more than 90% of their time underwater [3–5]. Describing sea turtle dive behavior is therefore a vital part of understanding sea turtle ecology and life histories.

The relationship between marine predator behavior and oceanographic conditions is not well understood [6]. However, some studies demonstrate that ocean temperature and foraging resources can interact and are important determinants for diving behavior. For example, elephant seals (*Mirounga leonina*) in the Southern Ocean dived deeper to forage and spent less time at those depths with increased water temperature [7], whereas Atlantic bluefin tuna (*Thunnus thynnus*) in the Mediterranean Sea dived deeper when biological productivity was high [8], and the depths reached in the North Atlantic for tuna were correlated with thermocline depth [9]. For sea turtles specifically, relationships between diving and interacting oceanographic conditions such as sea surface temperature (SST) and foraging resources have rarely been tested, although a few studies point to the potential importance of these factors. For olive ridleys (*Lepidochelys olivacea*) in the Guiana basin, foraging depth increased with a deeper thermocline and foraging time increased with lower temperatures [10] and leatherbacks (*Dermochelys coriacea*) in the Northwest Atlantic showed shorter, more shallow dives in cooler, productive shelf habitat [11]. While all of these species show variety in the appropriate temperature and depths for foraging based on their physiological requirements, these studies all show the importance of the interplay of ocean temperature and prey resources with diving.

A 2014 review on sea turtle dive behavior provides a summary of the fundamental knowledge gained on this important aspect of sea turtle life history [2]. From this review, we know that most sea turtle dive data have been collected on nesting females in neritic inter-nesting habitats, followed by juveniles in neritic developmental habitats. The deepest diving sea turtle is the leatherback which can reach 1250 m depth, while the record for the longest dive goes to loggerheads (*Caretta caretta*) in the Mediterranean at more than 10 h. For many hard-shelled sea turtles, depths visited on average (i.e. outside of overwintering) range from 2–54 m; for leatherbacks this ranges up to 150 m. The effect of temperature on sea turtles has been explored thoroughly and is shown to influence turtle metabolic rates, circulation and other physiological factors. Therefore, dive behavior is presumed to shift based on needs for thermoregulation and in response to seasonal changes (longer dives with lower temperatures), although across species and regions the relationship between temperature and diving has differed and was only investigated in 12 of 70 studies reviewed. The review also describes that some turtles change dive behavior based on whether they are transiting. For example, turtles tend to use shallow waters during transit, with occasional deep dives possibly for resting or foraging en route, with the exception of the leatherback that showed longer and deeper dives during transit. Importantly, dive behavior differed based on habitat type and geography. With all this variability, and an assumption that prey distribution is an important driver for sea turtle dive behavior, studies of dive characteristics alongside real-time prey vertical distribution is listed an important objective [2]; such studies can be challenging to undertake in the open ocean.

Loggerhead sea turtles (*Caretta caretta*) that nest on Gulf of Mexico (GoM) beaches represent three distinct populations segments (DPS [12]) within the threatened Northwest Atlantic population. The Northern Gulf of Mexico (NGoM) nesting group comprises one of the smallest DPSs, estimated at 323–634 adult females [13]. Loggerheads that nest in the NGoM appear to rarely leave the GoM [14, 15]. Although these individuals rely heavily on the GoM throughout their lives, very few dive data are available for turtles in this DPS.

Few studies have reported dive data for any loggerheads using GoM waters, except to describe basic depths reached (5 females [16]), dive durations (4 unsexed turtles [3]), time spent at the surface versus bottom (10 females [17]), or to show dive behavior during an extreme weather event (2 inter-nesting females [18]). While these studies contribute to our understanding of loggerhead dive behavior in this region, only one study covered turtles in this DPS [17]. Also, the studies did not investigate both depths and durations of dives across inter-nesting, migration and foraging and relate this to the environmental conditions of sea surface temperature and net primary productivity.

Using 26 tracks from loggerhead turtles that nested on NGoM beaches tagged with satellite-linked depth loggers, we provide new data in a region in which there is a minimal amount of dive data for this species and aim to investigate if and how oceanic conditions influence loggerhead dive behavior. Specifically, we assess the environmental variables of sea-surface temperature (SST) and net primary productivity (NPP). Using state-space modeling, we also determine the behavioral mode of each turtle (i.e. inter-nesting, migration, or foraging) along its track to determine how dive behavior and its relation to oceanographic conditions changes across the three major life stages of adult turtles.

## Materials and methods

### Loggerhead turtle tagging and dive data collection

**Fig 1:**
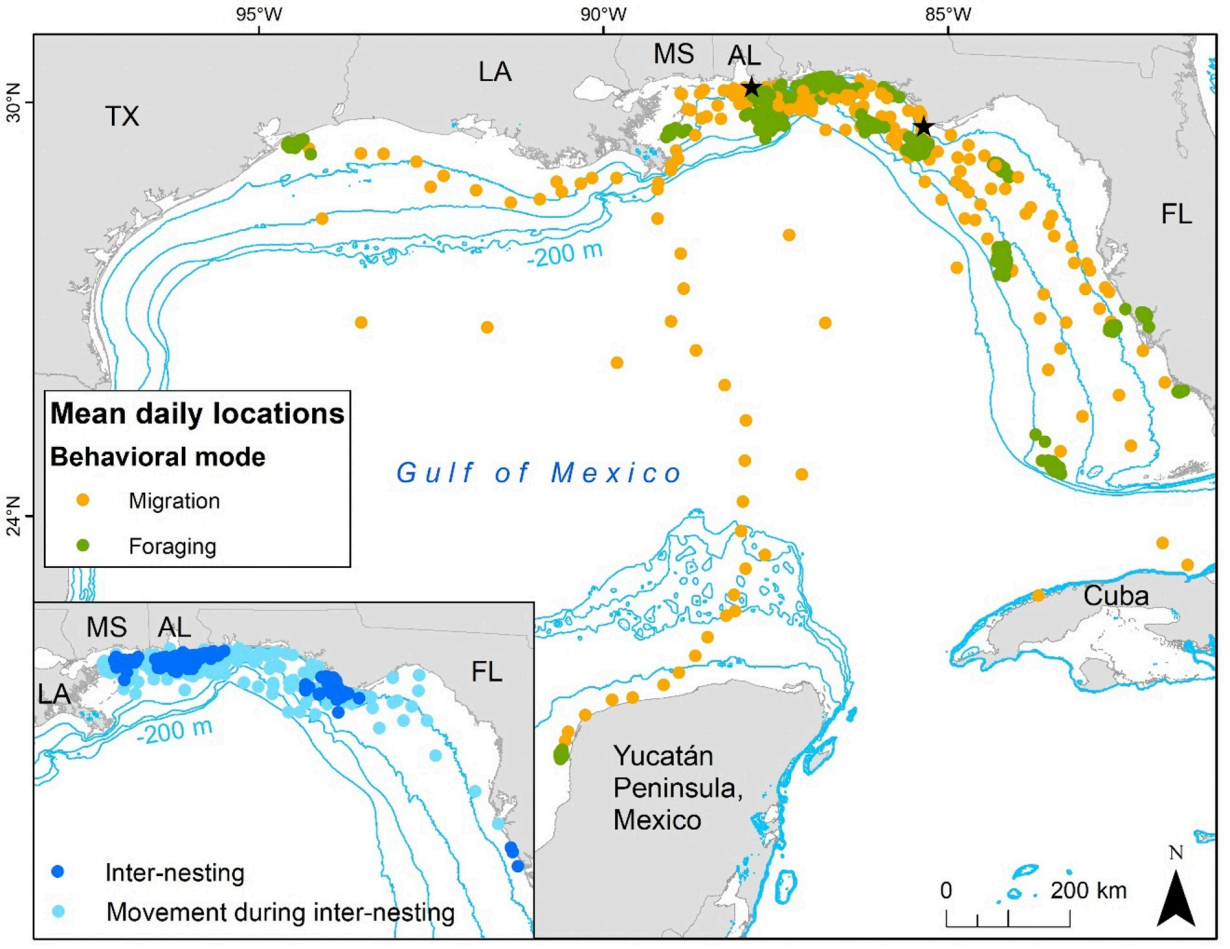
Mean daily locations (MDLs) of 26 adult female loggerhead turtles (*Caretta caretta*) tracks color-coded by behavioral mode. Main panel: migration and foraging MDLs; inset: inter-nesting and transit during inter-nesting MDLs (not all switching state-space model -defined transit periods were true migrations; see methods for full explanation of modes). Nesting beaches where turtles were tagged are shown with black stars and include from left to right: Gulf Shores, Alabama (AL) and St. Joseph Peninsula, Florida (FL). Contour lines indicate bathymetry at -200 m (furthest from land), -100 m, -50 m, and -20 m (closest to land).

We tagged turtles on NGoM beaches at Gulf Shores, Alabama (GS) and St. Joseph Peninsula, Florida (SJP), which are located in the eastern (SJP) and western (GS) ranges of loggerhead turtle nesting in the NGoM ([12]; Fig 1). Turtle capture and tagging followed methods identical to those in previous studies [19], followed established protocols [20] and were approved by Institutional Animal Care Protocol United States Geological Survey-Southeast Ecological Science Center-Institutional Animal Care and Use Committee-2011-05. We adhered platform transmitter terminals (PTTs) using slow-curing epoxy (two-part Superbond epoxy) and used two types of Wildlife Computers tags: SPLASH10-309A (n = 10; 7.6 cm long x 5.6 cm wide x 3.2 cm high, mass 125 g in air) and SPLASH10-238A-AF (n = 16; 10.5 cm long x 5.6 cm wide x 3.0 cm high, mass 213 g in air). Tags were set to collect dive data and transmit through the Argos satellite system for 24 hours day^-1^ all year, except tags with PTT identification numbers beginning with “129” (n = 10), which collected every day but transmitted every day from May-October then every 3rd day from November–April.

Histogram bins for dive duration (min) for all tags (n = 25, one tag used for two turtles) were set at 1, 2, 3, 4, 5, 10, 15, 20, 25, 30, 40, 50, 60 and >60. Dive depth (m) histogram bins were set at 5, 10, 15, 20, 25, 30, 35, 40, 45, 50, 100 and >100. Time-at-Depth (TAD; percent time within depth bins) histogram bins were set at 0, 1, 2, 3, 4, 5, 10, 20, 30, 40, 50, 100, 150, and >150 m. Dive data were summarized for every 24 h period, starting at 5:00 GMT (midnight local time), with two exceptions: tag 119947 summarized data every 12 h (local time: 0:00–12:00 and 12:00–0:00) and tag 119949 started summary periods at 4:00 GMT (23:00 local time). All tags also reported the maximum depth reached per day and time-at-temperature; GPS data was collected for all tags except those with PTT identification numbers beginning with “129.” We set tags to ignore dives shallower than 1 m and shorter than 30 s, which applied to depth and duration bins. We defined dives in the 1 min duration bin and in the 0–1 m TAD bins as “surface.”

### Location filtering and behavioral modes

Location data were retrieved using Satellite Tracking and Analysis Tool (STAT; [21]) available on www.seaturtle.org. We used switching state-space modeling (SSM) output from previous analyses [14], which included all received locations except those with Location Class “Z” which are classified as invalid. Briefly, SSM takes irregular locations received from satellites and performs a two-state switching correlated random walk to model transitions between behavioral states as well as “true” unobserved locations at equal time intervals [22], in this case, every eight hours. Behavioral mode output was binary, defined based on whether the turtle demonstrated area-restricted searching (i.e. ‘foraging and/or inter-nesting’) or not (transiting, i.e. ‘non-migration transit and/or migration’).

We defined points as ‘inter-nesting’ if they were before migration and ‘foraging’ if after. Because some turtles had multiple SSM transit periods, we considered two factors in order to discriminate migrations from non-migration transit: beach encounters of nesting turtles and graphs of the displacement distance from tagging locations [23]. If a nesting event (ground-truthed during nesting surveys) fell within the SSM-defined migration period, we classified the locations before the nest as ‘transit within the inter-nesting period’ (see [24]) and locations from the nesting date to the arrival at the foraging grounds as migration. We sub-divided ‘transit and migration’ into three categories: non-migratory transits within the nesting season, migration from the nesting grounds to foraging areas, and non-migratory transit within the foraging area (See S1 Fig for an example track showing mode designations).

For a few turtles, we had to make decisions beyond the above steps to determine behavioral mode. Turtle 106358 did not have a successful SSM run; locations for this turtle became very erratic and transmitted infrequently after August 13, 2011, after which high-quality locations occurred on the Cuban shoreline with subsequent locations moving inland. For our analysis, we only used locations before this date. We visually assessed the track to assign behavioral mode; the turtle moved multiple times between GS and SJP beaches and visited the shore often, indicating that the entire period was within an inter-nesting period, with some points making obvious directed movement classified as transits during inter-nesting. Additionally, for three turtles (129513, 106337, 119946), data stopped transmitting in August or early September of the tagging year (Aug 12 and 31, Sep 4 respectively), and their final area-restricted search areas were close to shore. The nesting season for this sub-population can occur through early September [25] making it unclear if these final areas should be considered inter-nesting or foraging grounds. To determine this, we used the average start of migration for the other turtles in this study (i.e. average end of nesting season: July 21st). Each turtle’s final period began either after this or only 4 days before (turtle 119946), and none had high-quality locations on land during this time, so we classified all three as foraging periods. This is also consistent with the peak in migration timing (July 22 –Aug 9) found for loggerhead turtles from this area (including some turtles from this study and others [14]).

With the beginning and end dates for different behavioral modes determined, we used original, filtered satellite locations from within those time periods for further spatial analysis. To filter the Argos locations, we removed points on land, those requiring speeds >5 kph, points with location class Z (those that failed Argos plausibility tests), and erroneous locations (defined as those outside the GoM). Because dive data does not come with a spatial location, we created points representing mean daily locations from filtered locations to associate dive data with corresponding behavioral modes and environmental data.

### Variables and data analysis

#### Environmental variables

To understand the importance of temperature and forage resources on loggerhead turtle diving, we used average monthly SST data and average monthly NPP data (as a proxy for food availability) in generalized linear mixed models (see below). We extracted SST and NPP data at mean daily locations and assigned a daily value for dive data. SST data was retrieved from NASA’s Ocean Color Web (https://oceancolor.gsfc.nasa.gov/, accessed on 8/5/2016 and 10/12/2016). NPP data was obtained from Oregon State University’s Ocean Productivity (https://www.science.oregonstate.edu/ocean.productivity/, accessed on 8/5/2016 and 10/17/2016).

#### Describing dives

Dive frequency may indicate behaviors such as resting or foraging, with longer dives at night indicating resting and shorter dives during the day indicating foraging [26, 27]. For each turtle and behavioral mode, we summed the number of dives day^-1^ for depth bins as well as duration bins; the number of dives in depth bins were used for analyses on daily frequency of dives (see modeling section below). Upper limits for “routine” diving (as opposed to maximum capabilities) in loggerhead turtles have been reported at 22 m depth and 30 min in duration [4]. Therefore, we defined “long” dives as those > 30 min and “deep” dives as those >25 m (our bin choices included either 20–25 m or 25–30 m).

We also summarized the proportion of time turtles spent in different Time-at-Depth (TAD) bins. Values in TAD bins represent the percent of time a turtle spent in a depth bin for the summary period (e.g. 24 hr). Using R [28], we averaged the values per bin across the entire tracking period for each turtle (using only full summary periods–any partial summary periods were removed). Then, we averaged these mean percentages across all turtles to get overall means and means per behavioral mode.

Additionally, each mean daily location was paired spatially with a bathymetry value and temporally with dive information. Any dives within the bin containing that bathymetry value (or deeper) were classified as dives to the bottom. We only evaluated depths up to 50 m because the bin sizes beyond 50 m (50–100 m and >100 m bins) were much larger than the other 5 m bin sizes. For bathymetry, we used the NOAA National Geophysical Data Center ETOPO1, 1 arc-minute global relief model of Earth’s surface (http://www.ngdc.noaa.gov/mgg/geodas/geodas.html; accessed 26 January 2012).

Finally, loggerhead turtles in the Mediterranean have shown a shift in dive behavior during winter including much longer dives [29, 30]. We examined possible overwintering behavior by describing diving during the winter (21 December– 21 March). For depth bins, duration bins and TAD bins, we used R [28] to create boxplots of the proportion of dives in each bin across all turtles.

#### Modeling dive behavior

To determine which factors influenced dive behavior, we took a model selection approach using four measurements of behavior: (1) total daily dive frequency, (2) frequency of dives to the bottom, (3) frequency of long dives and (4) Time-at-Depth focused on the time at surface. We chose three independent variables: NPP, SST, and mode (inter-nesting, foraging, migration). For all models, we combined inter-nesting and transit during inter-nesting dives (and defined both as inter-nesting) as turtles were in similar spatial locations, dives occurred over similar time periods (nesting season) and they showed similar depth patterns (maximum of individual median max depths for inter-nesting: 27.1 m and for inter-nesting transits: 32.6 m). For each of the four dive measurements, we tested 15 models, including a null model (intercept model) and one to three variable models including various interaction terms between SST, NPP and MODE. We used a generalized linear mixed model with a Poisson log-link function, with ‘individual turtle’ as a random effect because observations for each turtle were repeated. We selected the best-fitting model among the 15 models for each dependent variable (four dive measurements) using pseudo Akaike Information Criteria corrected for small sample sizes (AICc). Pseudo AICc was used because we did not estimate the model parameters using ordinary least squares. We considered models in which ΔAICc < 2 to be equivalent to the best-fitting model [31]. We also calculated the pseudo AICc weight, which represents a relative likelihood of the model.

## Results

### Turtles and behavioral modes

From 2011 to 2013, we tagged and successfully received dive data for 25 adult female nesting loggerhead turtles for a total of 26 tracks (n = 19 from GS, n = 7 from SJP). Two tracks (119944 and 106345) were from the same turtle tracked in subsequent years (2011 and 2012 in GS) and two turtles shared the same tag (119952a and 119952b in 2012) but for different time periods. From here on, 119944 and 106345 are treated as separate individuals but “individual” was taken into effect for all models. Turtles ranged in size from 87.3–104.3 cm CCL (mean ± SD = 95.9 ± 4.6 cm; n = 26) and were tracked from 23–404 days (mean ± SD = 122.7 ± 89.2 days; n = 26; S1 Table). Tracking produced a total of 1753 mean daily locations (Fig 1). Across all possible 3165 tracking days, daily summary periods for dive data were received for 2061 (dive depth, 62%), 1987 (dive duration, 60%) and 1992 (time-at-depth, 60%) days. Location and dive data were coincident on 1227 days. The total number of days in each mode ranged from 4 to 1810 and the total mean daily locations ranged from 4 to 966 (S2 Table).

All turtles remained in the GoM; inter-nesting and foraging locations were within the neritic zone (0 to -200 m bathymetry) ranging from Texas to southwestern Florida and migration locations were within and outside the neritic zone ranging from Texas to the Yucatan Peninsula and northern Cuba (Fig 1). Out of 26 tracks, eight had dive data in the southern GoM (n = 1 for inter-nesting, n = 7 for migration and n = 5 for foraging). Dive parameters by individual are listed in the S3 Table. Tracking details and home range analyses for some of these turtles can be found in previous papers [14, 19].

### Environmental variables

For all 1227 days/locations we were able to derive bathymetry and SST information, but NPP data was only available for 1003 locations. We filtered out 17 locations with SST values of 45.0°C as these locations were very close to shore and we considered the temperatures unrealistic as satellites may have been picking up terrestrial heat signals. For the remaining 1210 locations, bathymetry values ranged from -3352 to 1 m (mean ± SD = -52.7 ± 258.0 m) and SST values ranged from 18.0–33.7°C (mean ± SD = 29.0 ± 2.2°C; S2 Fig). The 1003 NPP values ranged from 144.6–6845.1 mg C square meter^-1^ day^-1^ (mean ± SD = 1763.5 ± 1162.4 mg C square meter^-1^ day^-1^; S2 Fig).

### Dive frequency

**Fig 2:**
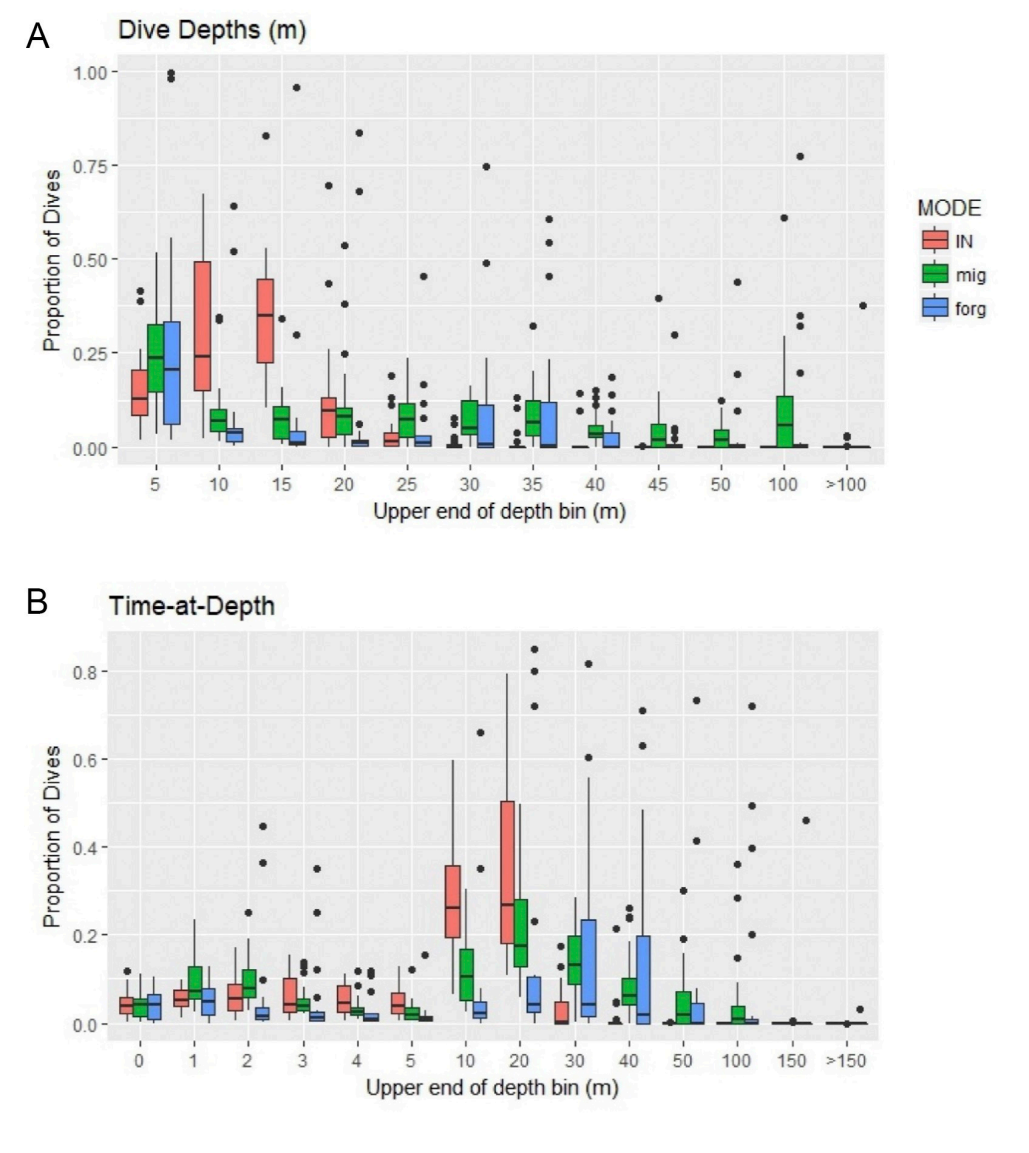
Boxplots for dive depth and the proportion of time-at-depth (TAD) for adult female loggerhead turtles (*Caretta caretta*) in the Gulf of Mexico. Proportions of dive depths (A) represent per turtle and per mode values with the dives per bin divided by the total dives for that mode. Values in TAD bins (B) represent the percent of time a turtle spent in a depth bin for the summary period (e.g. 24 hours). We then averaged the values per bin within dates for each behavioral mode.

Most dives were shallow (less than or equal to 25 m; Fig 2A). The overall median max depth for behavioral modes ranged from 10.3–27.1 m during inter-nesting (n = 21), 16.0–75.0 m during migration (n = 21), and 3.8–120.0 m during foraging (n = 19). The deepest dive recorded was 160 m; this was recorded for two separate turtles, one during migration (119945, middle of GoM) and one during foraging (119952, off AL coastline).

With all data combined, the mean total dive frequency was 41.9 dives per day (SD = 20.3). There was a large individual variability in total dive frequency. The largest mean dive frequency was 79.4 times per day (SD = 57.4) recorded by the second largest individual (119950; 102 cm CCL). This turtle also recorded the maximum daily dive frequency of 183 times. The lowest mean dive frequency was 27.2 times per day (SD = 16.6), recorded by a relatively small-sized turtle (106361; 92 cm CCL).

**Fig 3:**
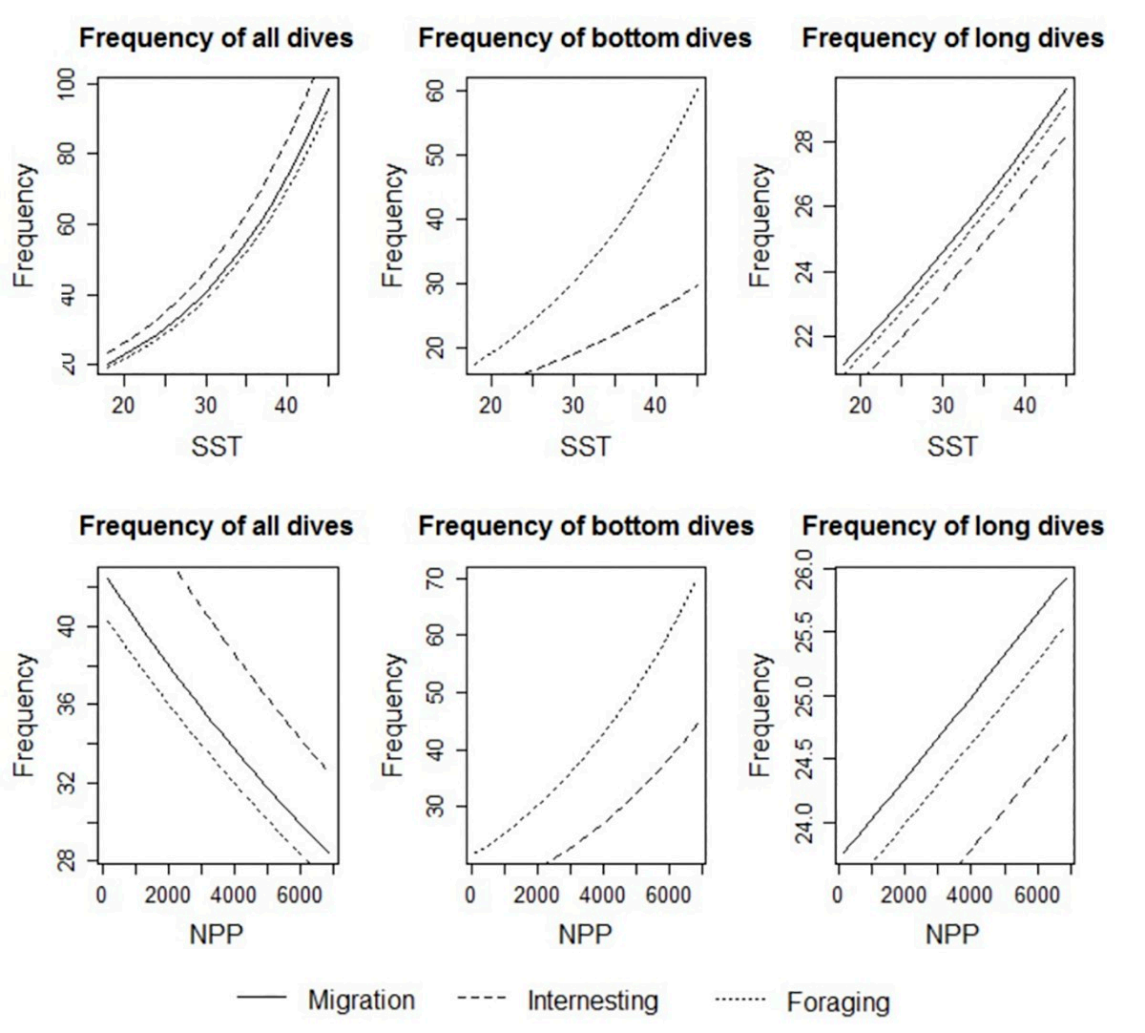
Prediction plots for dive metrics in relation to sea surface temperature (SST) and net primary productivity (NPP) for adult female loggerhead turtles (*Caretta caretta*) for each behavioral mode (migration, foraging and inter-nesting) in the Gulf of Mexico. Dive metrics include frequency of all dives per day, frequency of bottom dives per day, and frequency of long dives per day. For SST plots, NPP was held constant at the average value (1,763 mg C square meter^-1^ day^-1^). For NPP plots, SST was held constant at the average value (29°C). Behavioral modes are indicated by differing dash patterns. Note the variable y-axis scales for each plot.

MODE, SST and NPP are the best explanatory variables for dive frequency which minimized AICc (S4 Table). This model has the definite likelihood (AICc weight = 1.00) to be the best model among all tested models. Based on this model, the dive frequency was lower during the foraging period compared to the migration period (Table 1; *t* = -2.83, p < 0.01) whereas dive frequency was higher during the inter-nesting period (*t* = 6.4, p < 0.01). Higher SST is associated with a larger number of dives (Table 1; *t* = 17.09, p < 0.01) as is lower NPP (*t* = -8.09, p < 0.01; Fig 3). While the rates of change vary depending on NPP and SST values, with NPP held at the average value, for each increase in SST by 1°C (from 29 to 30°C) there was an increase of 2.3, 2.7, and 2.2 dives per day respectively for migration, inter-nesting and foraging (S5 Table).

**Table 1:** Estimated parameters for the best models for behavioral variables.

Variable	Estimate	Standard error	*t*-value	p
**Frequency of all dives day**^-1^				
Mode (inter-nesting)	0.1355	0.02116	6.4	< .001
Mode (foraging)	-0.05332	0.01882	-2.83	0.0047
SST	0.05861	0.003429	17.09	< .0001
NPP	-0.00006	6.82E-06	-8.09	< .0001
**Frequency of bottom dives day**^-1^				
Mode (inter-nesting) x SST	0.02992	0.004566	6.55	< .0001
Mode (foraging) x SST	0.04550	0.004420	10.29	< .0001
NPP	0.000173	0.000010	17.42	< .0001
**Frequency of long dives day**^-1^				
Mode (inter-nesting)	-0.0492	0.02226	-2.21	0.0273
Mode (foraging)	-0.1147	0.02117	-5.42	< .0001
SST	0.0124	0.002601	4.77	< .0001
NPP	0.000013	1.56E-06	8.4	< .0001

P-values and t-values are from tests on whether each coefficient is different from zero and indicate the significance of each effect in the models. For behavioral mode, migration mode was used as the reference category for all analyses except for the analysis of frequency of bottom dives in which foraging mode was used as the reference category.

### Dives to the bottom

For all inter-nesting and foraging periods combined, turtles dived to the bottom at least once on 95.5% of days (715/750 days). The mean frequency of daily bottom dives per turtle ranged from 3.4 to 82.3 times. At least one bottom dive was recorded on 96.9% of days (253/261 days) during inter-nesting and on 95% of days (463 of 489 days) during foraging.

When we examined the dive frequency only during foraging periods, MODE, SST and NPP (with an interaction term for MODE and SST) are still the best explanatory variables for the frequency of dives to the bottom, which presumably represents foraging behavior (S4 Table). The tagged turtles tended to dive to the bottom more frequently under higher SST (foraging: *t* = 10.29, p < 0.01; inter-nesting: *t* = 6.55, p < 001) and higher NPP (*t* = 17.42, p < 0.01; Fig 3). The rates of change vary depending on NPP and SST, but with NPP held at the average value, for each increase in SST by 1°C (from 29 to 30°C) there was an increase of 0.56 and 1.35 bottom dives per day respectively for inter-nesting and foraging (S5 Table).

### Frequency of long dives

**Fig 4:**
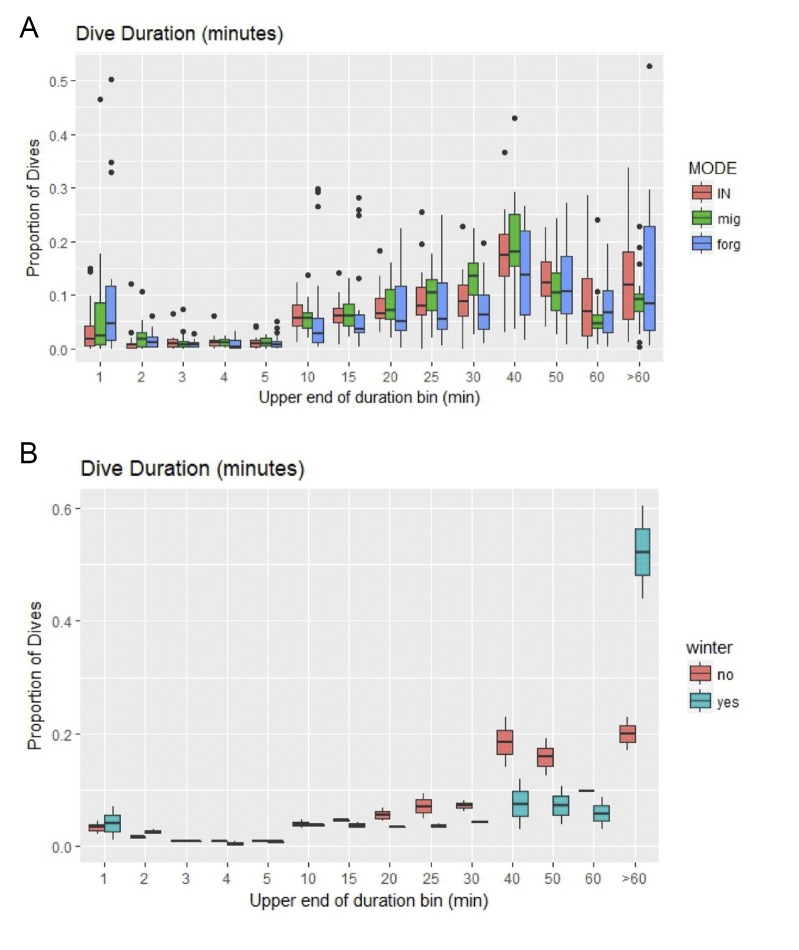
Boxplots for dive duration histograms for adult female loggerhead turtles (*Caretta caretta*) in the Gulf of Mexico. Proportions of dive durations (A) represent per turtle and per mode values with the dives per bin divided by the total dives for each mode. The lower panel (B) shows the proportions for the two turtles that had dive information in the winter. Values are only for foraging periods and proportions were created for each bin for dives in winter (“yes”, 21 December– 21 March) or not (“no”).

During all modes, long dives (> 30 min) and short dives occurred at about the same rate. The frequency of dives was highest in the 40 min bin (30–40 min) for all three modes, but there was also a peak in the >60 min bin for foraging mode (Fig 4A). For two turtles tracked over winter (21 December—21 March; turtles 119944 and 106361), we found about half of their winter dives occurred in the >60 min bin (Fig 4B).

The frequency of long dives was best explained by the three-variable model which included MODE, SST, and NPP (S4 Table). Compared to the migration periods, turtles tended to dive for long periods less frequently during inter-nesting (*t* = -2.21, p = 0.03) and foraging (*t* = -5.42, p < 0.01) periods. Frequency of long dives significantly increased when both SST (*t* = 4.77, p < 0.01) and NPP (*t* = 8.4, p< 0.01) were higher (Table 1; Fig 3). As before, the rates of change vary depending on NPP and SST values; with NPP held at the average value, for each increase in SST by 1°C (from 29 to 30°C) there was an increase of 0.30, 0.29, and 0.30 long dives per day respectively for migration, inter-nesting and foraging (S5 Table).

### Time-at-depth

Turtles spent most of their time between the surface and 30 m. Across all modes, turtles spent 10% of their time at the surface (0–1 m; by mode: 10% in inter-nesting, 14% in migration, 9% in foraging). Time in the deepest bins was rare with only 2% of time in the 100–150 m bin and less than 1% in the >150 m bin (Fig 2B). Across modes, turtles spent the highest proportion of time between 5 and 20 m during inter-nesting and migration, and between 20 and 40 m for foraging (Fig 2B).

TAD showed a deviation during winter from the rest of foraging behavior. Instead of time spent about equally between the three bins covering 10–40 m, in winter, this shifted to 26% (106361) to 68% (119944) of the time being spent between 30–40 m with much less time in the 10–30 m categories.

The results indicated that none of the tested variables sufficiently explain the TAD variables for surface dives; the null model minimized AICc for all TAD variables (S4 Table).

## Discussion

Understanding how sea turtles use three-dimensional space and what factors are important to the expression of dive behavior is key to understanding sea turtle life histories. Our dataset and comprehensive look at dive behavior for 26 adult female loggerhead turtle satellite tracks within the northern GoM significantly adds to what is known for loggerhead diving in this region. This is the first loggerhead dive study in the GoM to assess what oceanographic factors drive dive behavior across different behavioral modes and one of the first to provide detailed dive information for this DPS (see [17] for surface/bottom time for 5 females). We found that loggerhead dive behavior varied with changing SST, NPP and behavioral mode.

### Dive behavior

The deepest dive for our tagged turtles in the GoM was to 160 m which falls within previously reported maximum dive depths for loggerhead turtles of >340 m, from off the coast of Japan [32]. While informative, maximum depths do not represent behavior for all individuals and/or for all times; therefore, it is also important to consider median or mean dive behavior. In her review of sea turtle diving, Hochscheid found mean dive depths for loggerheads ranged from 5.2 to 54 m [2]. The proportion of number of dives for our tagged turtles, considering all modes, peaked in the 5 and 15 m bins and turtles spent little time deeper than 50 m, which is consistent with these reported mean values. Hochscheid also reported maximum dive durations for loggerheads from 4.8 to 614.4 min and mean or median dive durations from 2.3 to 341 min [2]. GoM loggerhead dive durations were also within these ranges (most dives in the 30–40 min bin across modes).

Overall, our turtles spent about 80% of their time between 0–30 m over their tracking periods. This is consistent with other studies looking at the proportion of time spent at depth. Two turtles from Japan spent most of their time between 0–25 m (0% and 35% of time at > 25 m depth [33]); six of seven loggerhead turtles from the Mediterranean (46–75 cm CCL) spent most of their time between 0–30 m [29]; and two loggerheads from the North Pacific (61–83 cm SCL) spent around 40% of their time within 1 m of the surface and almost no time deeper than 100 m [34].

For loggerhead turtles in the northern GoM, most dive behavior is conducted within the first 30 m of the water column. Foraging behavior has been described as consisting of a higher frequency of dives during the day with shorter durations as compared to night dives [26, 35]. Also, it would be expected that loggerheads rest at 20 m or shallower based on lung-regulated neutral buoyancy [36, 37]. We were unable to separate day and night diving because of tag settings, and so could not distinguish resting from foraging dives. Whether these turtles are choosing foraging and resting locations based on factors like distance to shore during inter-nesting or prey distribution during foraging, or whether bathymetry itself plays an important role in how they choose a location for each mode is unclear. Using fine-scale data loggers that provide dive profiles or accelerometer data, instead of binned data, could help determine what behaviors turtles are engaged in at depth, such as resting or foraging.

Two turtles tracked over winter showed an increase in longer dives (>60 min) to depths between 30–40 m. Maximum dive durations from other studies indicate that loggerhead turtles can spend long periods under water and the longest duration (614.4 minutes) recorded was for a loggerhead in the Mediterranean during the winter [38]. Sea turtles exhibit metabolic depression at lower temperatures thereby slowing use of oxygen reserves and allowing turtles to remain aerobic during long dives [39, 40]. Although it has been suggested that sea turtles in some locations remain dormant (i.e. hibernate) at temperatures below 10°C [41, 42], recent studies suggest an alternative; that turtles undertake long dives paired with infrequent surfacing events during winter [2, 43]. Unfortunately, the tag settings for our GoM data topped out at a bin value of > 60 min so it is unclear if these turtles stayed under for hundreds of minutes at any time, however other loggerheads in the GoM stayed under for > 4 hr during winter [44]. Future studies with more turtles and a wider range of dive duration bins or dive profile data instead of binned data could help determine the extent to which loggerheads in the GoM display over-wintering behavior; profiles provide more detailed information for individual dives (e.g. [45]). Additionally, change-point analysis incorporating dive data with location data to determine behavioral mode has recently helped predict a shift into wintering behavior for loggerheads in the Mediterranean [30].

### SST, NPP, and behavioral mode

Of 70 sea turtle dive studies reviewed, only 12 addressed the relationship between temperature and diving [2], despite the obvious importance of temperature in sea turtle physiology. For loggerheads in particular, studies have generally found longer dives in colder temperatures [29, 32, 46] and deeper dives in warmer temperatures [5, 32].

We found that SST was an important factor for predicting the frequency of (1) diving, (2) diving to the seafloor, and (3) long dives. Overall, higher SST was associated with more frequent diving. Increasing temperatures could affect turtles both directly and indirectly. For example, it is possible that because turtles are ectotherms, they are simply more active in warmer waters, or they use differing water temperatures for thermoregulation [2]. However, in all models, NPP was also an important predictor for dive behavior, indicating that SST alone does not drive dive behavior of sea turtles. While the idea that SST can indirectly affect sea turtle dive behavior through prey distribution is not new [2], our results explicitly showed that NPP was an important predictor for adult loggerhead dive behavior.

As adult loggerhead turtles primarily eat benthic invertebrates [47], we used NPP as a proxy for food availability. Most primary production in the southwestern GoM eventually becomes detritus and benthic invertebrates play a large role in consuming it, consequently making it available for higher trophic levels [48]. We found that higher NPP was associated with an increase in the frequency of long dives and the frequency of bottom dives. With more NPP (and presumably more food available), this behavior could reflect foraging behavior where turtles are spending longer periods of time foraging for prey on the seafloor. Lower NPP was associated with more dives per day overall, which could be a result of more searching in areas with less productivity.

Due to possible shifts in dive behavior over time, comparisons between specific behavioral modes may be more informative than broad comparisons across entire tracking periods, and our results support this as we found significant differences in diving across modes. Notably, for the frequency of bottom dives, the top model included an interaction between behavioral mode and SST, indicating that changes in diving with SST are ultimately dependent on the behavior a turtle is primarily engaged in, such as transiting, foraging and/or resting. Long dives occurred less often during inter-nesting or foraging as compared to migration, which reflects that turtles dive for > 30 min more often, perhaps either resting or swimming, during migration. Occasional very deep dives during transit phases for juvenile loggerheads were found in the Indian Ocean [27], with speculation that this could be due to prey searching, predator avoidance or navigation. We found similar behavior, as the majority of dives deeper than 45 m took place during migration. More long dives during migration may be a result of an increase in much deeper dives. However, this result may also be due to a lack of observations during winter at foraging grounds when longer dives would be expected and were indeed observed for the two turtles tracked into winter.

### Conservation and management

GoM loggerhead turtles spent around 10% of time at the surface (up to 1 m). To complement nest numbers, visual population counts of turtles at in-water sites is important to estimate the true number of turtles, including males which are not counted on the beach. Counting sea turtles visually, either from ships or during aerial surveys, is an important component to National Marine Fisheries Survey (NMFS) population assessment goals for sea turtles (www.st.nmfs.noaa.gov). This dive information can be used for aerial correction factors for NMFS as they 'correct' for a turtle’s time below the surface during their aerial surveys [49, 50]. Visual population estimates rely on accurate knowledge of the proportion of time that turtles spend in the top portion of the water column. The current Gulf of Mexico Marine Assessment Program for Protected Species (GoMMAPPS; https://www.boem.gov/GOMMAPPS/) will utilize time at surface information from these and other turtles to correct aerial survey point counts of turtles.

Dive data are also important for deciphering how and where turtles may interact with anthropogenic factors, which may pose multiple threats to turtles in the GoM [51]. Commercial fishing occurs throughout the GoM, especially in neritic areas where loggerhead turtles take up residence [51]. For example, loggerhead bycatch in the GoM bottom longline fisheries has been a concern, and studies show loggerheads overlap with these fishing areas, which present the threat of hooking or entanglement on the seafloor [16]. Knowing which depths turtles use most in each season or behavioral mode can help inform policy decisions for managers looking to reduce bycatch and conserve turtle populations. In this study we found that during inter-nesting and foraging modes, turtles dived to the bottom almost every day. The information we present here on the depths used by loggerheads during inter-nesting, migration and foraging mode should therefore be useful for local management decisions where loggerheads and bottom longline fisheries overlap.

### Conclusion

We found that SST and NPP were important for all the dive behaviors we measured. The degree to which changes in SST and NPP will affect the biology of sea turtles will depend on many factors beyond the number of additional dives, such as the depth and duration of additional dives (i.e. the energetic cost of each dive), the success in capturing prey, the type and density of prey consumed, and the water temperature both at the surface and at depth. Ultimately the physiological cost of each foraging dive will be a balance between energy expended and energy consumed in the form of prey. The changes in dive behavior we found across SST and NPP values may reflect shifting strategies that keep net energy intake relatively constant. However, we were limited in our ability to decipher what behavior turtles were engaged in at depth (e.g., resting or foraging), and had no knowledge of prey consumed, so we are unable to determine if changes in diving represent shifting strategies or an increased physiological cost. The behavioral mode behind each dive must also be considered. For example, during inter-nesting, loggerhead turtles may remain at certain depths to save energy for egg maturation [52] and so energy trade-offs during this time may have little to do with prey availability and more to do with temperature. To understand costs associated with different behaviors and additional dives, accelerometer data on sea turtle energetics during dives [53] could be linked to oceanic conditions.

Individual turtle variability likely also plays an important role in diving behavior which may partially explain why SST, NPP and behavioral mode did not successfully explain time spent at the surface. Surfacing behavior can vary depending on turtle activity; for example, green turtles (*Chelonia mydas*) took less surface breaths after foraging than resting, indicating that they modified their surfacing behavior based on the goal of the dive [54] and loggerheads may alter surface times to absorb solar radiation or recover from long dives [5]. Deeper dives may also show variability by individual. From video obtained through remotely operated vehicles, some loggerheads (2 of 73 filmed) in the U.S. Mid-Atlantic were found to feed on pelagic gelatinous prey before diving to the sea bottom, while others only fed at the benthos [55]. Stable isotope studies on 15 loggerheads sampled on the east coast of Florida, show that individual loggerheads specialize; however, considered together, the group were generalists [56]. The variation in prey choices across adults, but also loggerhead life-stages [57], would affect both the energetic gains received from prey and the costs in capturing it, both regarding diving depths and durations as well as the difficulty of capture.

Even without the knowledge of specific energetic costs of these additional dives on the turtles, establishing a link between loggerhead diving and both SST and NPP may have important ramifications. Prey availability is important for turtles but difficult to measure. Here we show that SST and NPP in the environment, both of which can be more easily measured than prey abundance, may be important for sea turtle biology. Further, how SST and NPP interact is of interest: global increases in SST are linked to declining ocean productivity [58] which may affect prey distribution and abundance and could ultimately cause turtles to show more searching and diving. Having baseline information on diving in relation to temperature and productivity could be helpful for detecting changes in sea turtle dive behavior as ocean surface temperatures are predicted to rise in the future [59].

## Supporting information

S1 Fig An example of the designation of behavioral mode based on tracking data and switching state-space model (SSM) output. This turtle (129508) was tagged in Gulf Shores, Alabama (AL) and the filtered locations from Argos are shown as grey X’s. After tagging, according to SSM it entered into a transit stage (peach circles) where it traveled to St. Joseph Peninsula, Florida (FL), and took up temporary residence (an area-restricted search inter-nesting stage; blue circles off the FL coast). The turtle then traveled back to the original nesting area (secondary transit during inter-nesting; peach circles) where it demonstrated another area-restricted search behavior (a secondary inter-nesting stage; blue circles south of AL). After this inter-nesting period, it then began transiting (migration; red triangles) to foraging grounds (area-restricted search; purple squares) where it remained from 18 August to at least 15 October. It is possible that this turtle stopped to nest again, and this went undetected; the turtle visited the FL coast twice on its journey to its foraging grounds however we were unable to confirm any nesting activity with high-quality locations or sightings on the beach.
(DOC)

S2 Fig Histograms of the sea surface temperature (SST, in ˚C) and net primary productivity (NPP, in mg C/m^2^/day) at mean daily locations across different behavioral modes (inter-nesting, migration and foraging) for adult female loggerhead turtles (*Caretta caretta*) in the Gulf of Mexico. (DOC)

S1 Table Adult female loggerhead turtles (*Caretta caretta*) satellite-tagged with depth loggers after nesting on Gulf of Mexico beaches. Tag Loc = nesting beaches where turtles were tagged include Gulf Shores, Alabama (GS) and St. Joseph Peninsula, Florida (SJP). CCL is curved carapace length.
(DOC)

S2 Table Summary of the number of turtles, periods, days and mean daily locations (MDLs) for each behavioral mode. Turtles = those with locations in the corresponding mode (out of 26 total). Periods = the total number of times turtles were in the corresponding mode. Days = total number of days across all turtles for that mode. MDLs = total mean daily locations for that mode. MDL does not equal Days because locations were not always received every tracking day.
(DOC)

S3 Table Size and dive parameters by individual for loggerhead turtles (*Caretta caretta*) in this study. CCL = curved carapace length, SCL = straight carapace length. IN = inter-nesting, t-IN = transit during IN, M = migration, t-F = transit during foraging, F = foraging; see Methods for how these behavioral modes were determined. “.” = not applicable or data not available. Dive durations were collected in bins, with the >60 min bin the largest bin set in tag settings.
(DOC)

S4 Table Dive behavior model results for adult female loggerhead turtles. For each dive behavior variable, the models are ordered by AICc from the smallest to the largest.
(DOC)

S5 Table The change in dive metric by behavioral mode in relation to sea surface temperature (SST) and net primary productivity (NPP). The change is calculated for both the coldest SST and average SST values recorded (over a 1°C change) and the lowest and average values of NPP (over a 500 mg change). Dive metrics include the frequency of all dives, the frequency of bottom dives and the frequency of long dives. For SST, NPP was held constant at the average value (1,763 mg C square meter-1 day-1). For NPP plots, SST was held constant at the average value (29°C). In the table "mg C" is measured per square meter per day.
(DOC)
