# Supplementary material for: Loggerhead sea turtle (Caretta caretta) diving changes with productivity, behavioral mode, and sea surface temperature
Source: PLoS One. 2019 Aug 7;14(8):e0220372. doi: 10.1371/journal.pone.0220372 (PMC6685635; doi:10.1371/journal.pone.0220372)
Supplement: S1 Fig — This turtle (129508) was tagged in Gulf Shores, Alabama (AL) and the filtered locations from Argos are shown as grey X’s. After tagging, according to SSM it entered into a transit stage (peach circles) where it traveled to St. Joseph Peninsula, Florida (FL), and took up temporary residence (an area-restricted search inter-nesting stage; blue circles off the FL coast). The turtle then traveled back to the original nesting area (secondary transit during inter-nesting; peach circles) where it demonstrated another area-restricted search behavior (a secondary inter-nesting stage; blue circles south of AL). After this inter-nesting period, it then began transiting (migration; red triangles) to foraging grounds (area-restricted search; purple squares) where it remained from 18 August to at least 15 October. It is possible that this turtle stopped to nest again, and this went undetected; the turtle visited the FL coast twice on its journey to its foraging grounds however we were unable to confirm any nesting activity with high-quality locations or sightings on the beach. (DOC) [file pone.0220372.s001.doc]

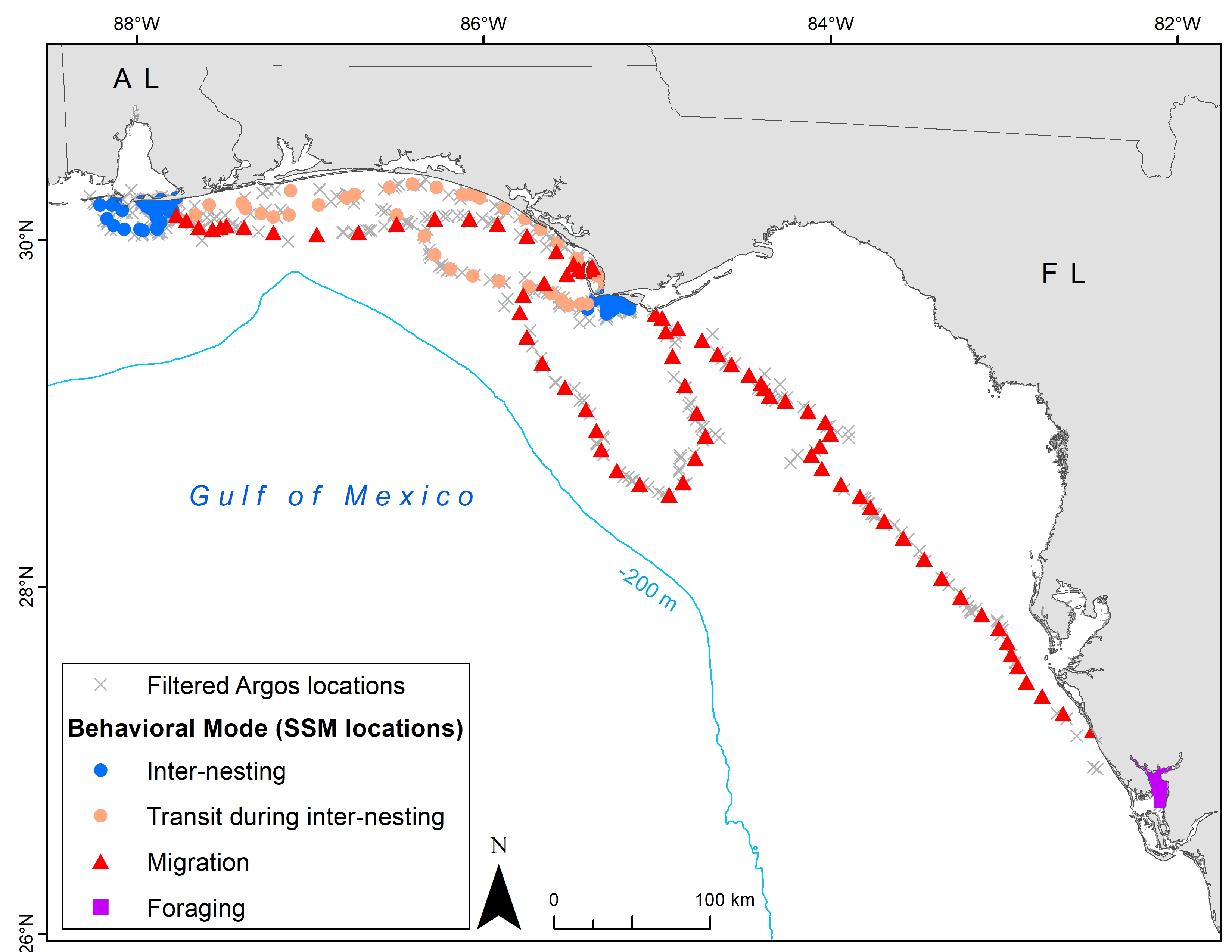


**S1 Fig. An example of the designation of behavioral mode based on tracking data and switching state-space model (SSM) output.** This turtle (129508) was tagged in Gulf Shores, Alabama (AL) and the filtered locations from Argos are shown as grey X’s. After tagging, according to SSM it entered into a transit stage (peach circles) where it traveled to St. Joseph Peninsula, Florida (FL), and took up temporary residence (an area-restricted search inter-nesting stage; blue circles off the FL coast). The turtle then traveled back to the original nesting area (secondary transit during inter-nesting; peach circles) where it demonstrated another area-restricted search behavior (a secondary inter-nesting stage; blue circles south of AL). After this inter-nesting period, it then began transiting (migration; red triangles) to foraging grounds (area-restricted search; purple squares) where it remained from 18 August to at least 15 October. It is possible that this turtle stopped to nest again, and this went undetected; the turtle visited the FL coast twice on its journey to its foraging grounds however we were unable to confirm any nesting activity with high-quality locations or sightings on the beach.
