# Supplementary material for: Loggerhead sea turtle (Caretta caretta) diving changes with productivity, behavioral mode, and sea surface temperature
Source: PLoS One. 2019 Aug 7;14(8):e0220372. doi: 10.1371/journal.pone.0220372 (PMC6685635; doi:10.1371/journal.pone.0220372)
Supplement: S2 Fig — (DOC) [file pone.0220372.s002.doc]

**
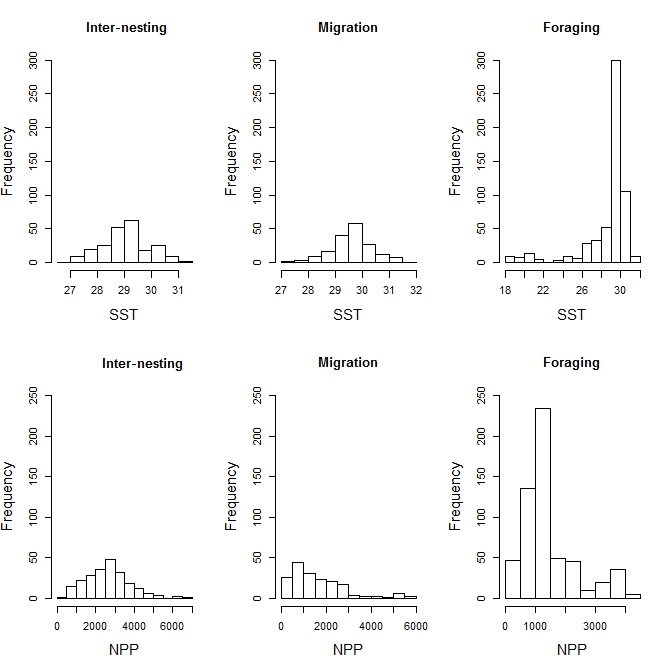
**

**S2 Fig. Histograms of the sea surface temperature (SST, in ˚C) and net primary productivity (NPP, in mg C/m^2^/day) at mean daily locations across different behavioral modes (inter-nesting, migration and foraging) for adult female loggerheads (*Caretta caretta*) in the Gulf of Mexico.**
