# Supplementary material for: Loggerhead sea turtle (Caretta caretta) diving changes with productivity, behavioral mode, and sea surface temperature
Source: PLoS One. 2019 Aug 7;14(8):e0220372. doi: 10.1371/journal.pone.0220372 (PMC6685635; doi:10.1371/journal.pone.0220372)
Supplement: S1 Table — Tag Loc = nesting beaches where turtles were tagged include Gulf Shores, Alabama (GS) and St. Joseph Peninsula, Florida (SJP). CCL is curved carapace length. (DOC) [file pone.0220372.s003.doc]

**S1 Table. Adult female loggerhead turtles (*Caretta caretta*) satellite-tagged with depth loggers after nesting on Gulf of Mexico beaches.**

| **Turtle** | **Tag Loc** | **CCL (cm)** | **Tracking period (days)** |
| --- | --- | --- | --- |
| 129506 | GS | 96.1 | 6/11 - 10/23/2013 (135) |
| 129507 | GS | 99 | 6/11 - 10/25/2013 (137) |
| 129508 | GS | 97.3 | 6/12 - 10/15/2013 (126) |
| 129509 | GS | 90.4 | 6/12 - 10/25/2013 (136) |
| 129510 | GS | 97 | 6/14 - 10/14/2013 (123) |
| 129511 | GS | 98 | 6/19 - 9/23/2013 (97) |
| 129512 | GS | 104.3 | 6/16 - 10/23/2013 (130) |
| 129513 | GS | 93.4 | 6/17 -8/12/2013 (57) |
| 129514 | SJP | 87.3 | 7/10 - 10/25/2013 (108) |
| 129515 | GS | 100.4 | 6/23 - 9/22/2013 (92) |
| 119943 | GS | 97.5 | 6/4 - 11/20/2012 (170) |
| 119944 | GS | 90.8 | 6/7/2012 - 7/15/2013 (404) |
| 119945 | GS | 98.9 | 6/9 - 8/8/2012 (61) |
| 119946 | GS | 95 | 6/9 - 9/4/2012 (88) |
| 119947 | GS | 98.5 | 6/13 - 8/12/2012 (61) |
| 119948 | SJP | 92.5 | 6/11 - 10/12/2012 (124) |
| 119949 | SJP | 100 | 6/11 - 7/20/2012 (40) |
| 119950 | SJP | 102 | 6/11 - 8/5/2012 (56) |
| 119951 | SJP | 103.3 | 6/11 - 8/2/2012 (53) |
| 119952a | SJP | 101.1 | 6/14- 7/6/2012 (23) |
| 119952b | SJP | 90.1 | 7/23 - 10/3/2012 (73) |
| 106360 | GS | 92.3 | 6/7 - 12/24/2011 (201) |
| 106345 | GS | 90.1 | 6/9 - 11/10/2011 (155) |
| 106337 | GS | 93.6 | 6/11 - 8/31/2011 (82) |
| 106358 | GS | 92.5 | 6/12 - 8/13/2011 (63) |
| 106361 | GS | 92 | 6/15/2011 - 6/18/2012 (370) |
| *mean* |  | *95.9* | *121.7* |
| *sd* |  | *4.5* | *89.2* |

Tag Loc = nesting beaches where turtles were tagged and include Gulf Shores, Alabama (GS) and St. Joseph Peninsula, Florida (SJP). CCL is curved carapace length.
