# Supplementary material for: Loggerhead sea turtle (Caretta caretta) diving changes with productivity, behavioral mode, and sea surface temperature
Source: PLoS One. 2019 Aug 7;14(8):e0220372. doi: 10.1371/journal.pone.0220372 (PMC6685635; doi:10.1371/journal.pone.0220372)
Supplement: S2 Table — Turtles = those with locations in the corresponding mode (out of 26 total). Periods = the total number of times turtles were in the corresponding mode. Days = total number of days across all turtles for that mode. MDLs = total mean daily locations for that mode. MDL does not equal Days because locations were not always received every tracking day. (DOC) [file pone.0220372.s004.doc]

**S2 Table. Summary of the number of turtles, periods, days and mean daily locations (MDLs) for each behavioral mode.**

| **Mode** | **Turtles** | **Periods** | **Days** | **MDLs** |
| --- | --- | --- | --- | --- |
| Inter-nesting | 21 | 33 | 612 | 330 |
| Transit during inter-nesting | 17 | 30 | 369 | 259 |
| Migration | 21 | 22 | 319 | 193 |
| Foraging | 19 | 21 | 1810 | 966 |
| Transit during foraging | 2 | 2 | 4 | 4 |

Turtles = those with locations in the corresponding mode (out of 26 total). Periods = the total number of times turtles were in the corresponding mode. Days = total number of days across all turtles for that mode. MDLs = total mean daily locations for that mode. MDL does not equal Days because locations were not always received every tracking day.
