# Supplementary material for: Loggerhead sea turtle (Caretta caretta) diving changes with productivity, behavioral mode, and sea surface temperature
Source: PLoS One. 2019 Aug 7;14(8):e0220372. doi: 10.1371/journal.pone.0220372 (PMC6685635; doi:10.1371/journal.pone.0220372)
Supplement: S3 Table — CCL = curved carapace length, SCL = straight carapace length. IN = inter-nesting, t-IN = transit during IN, M = migration, t-F = transit during foraging, F = foraging; see Methods for how these behavioral modes were determined. “.” = not applicable or data not available. Dive durations were collected in bins, with the >60 min bin the largest bin set in tag settings. (DOC) [file pone.0220372.s005.doc]

**S3 Table. Size and dive parameters by individual for loggerhead turtles (*Caretta caretta*) in this study.**

| **Turtle** | **SCL-tip (cm)** | **CCL-tip (cm)** | **Maximum Dive Depth (m)** | **Max Dive Duration (min)** | **IN max depth range (m)** | **IN median max depth (m)** | **m-IN max depth range (m)** | **m-IN median max depth (m)** | **M max depth range (m)** | **M median max depth (m)** | **m-F max depth range (m)** | **m-F median max depth (m)** | **F max depth range (m)** | **F median max depth (m)** |
| --- | --- | --- | --- | --- | --- | --- | --- | --- | --- | --- | --- | --- | --- | --- |
| 129506 | 88.5 | 96.1 | 76.8 | >60 | 8.5-17.5 | 14.8 | 8.75-31.25 | 15.8 | 15.75-73.5 | 50.8 | . | . | 45-76.75 | 65.5 |
| 129507 | 89.6 | 99.0 | 106.8 | >60 | 10-30.5 | 17.1 | 7-42.5 | 17.3 | 8-106.75 | 56.5 | . | . | 8-16.5 | 9.5 |
| 129508 | 89.1 | 97.3 | 50.0 | >60 | 9.25-22.25 | 13.8 | 10-47.5 | 24.5 | 9.0-50 | 28.5 | . | . | 1.75-6.25 | 3.8 |
| 129509 | 82.8 | 90.4 | 71.0 | >60 | 0.375-25.25 | 12.5 | 3.25-24.75 | 16.3 | 30.5-71 | 47.4 | 32.5-37.5 | 35.0 | 20.25-37.5 | 27.8 |
| 129510 | 90.0 | 97.0 | 71.0 | >60 | . |  | 9-63.5 | 25.3 | 27.5-71 | 44.9 | . | . | 42.75-47.5 | 47.5 |
| 129511 | 87.9 | 98.0 | 77.8 | >60 | 7.5-17.5 | 12.9 | 4.25-77.75 | 28.3 | 56.5-65.75 | 63.0 | . | . | 45-75 | 50.5 |
| 129512 | 95.0 | 104.3 | 27.5 | >60 | 0.75-27.5 | 15.8 | . | . | 15.75-19.5 | 17.0 | . | . | 7.5-22.5 | 19.3 |
| 129513 | 86.0 | 93.4 | 67.5 | >60 | 10.5-17.25 | 15.5 | 3.75-34.25 | 17.0 | 15.25-67.5 | 29.4 | . | . | 12.25-15.75 | 12.8 |
| 129514 | . | 87.3 | 42.5 | >60 | 19.5-32.5 | 27.1 | 16.25-37.5 | 32.6 | 12-42.5 | 28.5 | . | . | 22.5-40.75 | 29.5 |
| 129515 | 94.7 | 100.4 | 62.3 | >60 | 5.0-25 | 15.8 | 5.25-28.25 | 17.5 | 15.25-62.25 | 33.3 | 32.5-33.25 | 32.9 | 27.75-37.5 | 30.8 |
| 119943 | 89.8 | 97.5 | 56.0 | >60 | . | . | 15-40 | 25.0 | 22.5-56 | 35.0 | . | . | 25-37.5 | 27.5 |
| 119944 | 81.6 | 90.8 | 56.0 | >60 | . | . | . | . | 12.5-37.5 | 19.3 | . | . | 25-56 | 40.0 |
| 119945 | 90.6 | 98.9 | 160.0 | >60 | . | . | . | . | 15-160 | 64.0 | . | . | . | . |
| 119946 | 89.0 | 95.0 | 80.0 | >60 | 7.5-15 | 10.3 | 7.5-27.5 | 22.5 | 22.5-80 | 37.5 | . | . | 4.5-17.5 | 7.5 |
| 119947 | 92.5 | 98.5 | 22.5 | >60 | 12.5-22.5 | 17.5 | . | . | . |  | . | . | . | . |
| 119948 | 85.2 | 92.5 | 37.5 | >60 | 7.5-25 | 12.5 | 12.5-12.5 | 12.5 | 2.5-37.5 | 16.0 | . | . | 2.5-8 | 4.0 |
| 119949 | 91.5 | 100.0 | 22.5 | >60 | 7.5-22.5 | 12.5 | 12.5-22.5 | 12.5 | . |  | . | . | . | . |
| 119950 | . | 102.0 | 42.5 | >60 | 7.5-42.5 | 17.5 | . | . | . |  | . | . | . | . |
| 119951 | . | 103.3 | 125.0 | >60 | 7.5-27.5 | 12.5 | . | . | 45-125 | 75.0 | . | . | . | . |
| 119952a | 92.9 | 101.1 | 40.0 | >60 | 7.5-16 | 12.5 | 7.5-40 | 27.5 | . |  | . | . | . | . |
| 119952b | 85.0 | 90.1 | 160.0 | >60 | 7.5-25 | 15.5 | . | . | 40-75 | 47.5 | . | . | 37.5-160 | 120.0 |
| 106360 | 88.3 | 92.3 | 80.0 | >60 | 7.5-17.5 | 12.5 | . | . | 16-80 | 64.0 | . | . | 45-80 | 64.0 |
| 106345 | 83.7 | 90.1 | 47.5 | >60 | 7.5-22.5 | 15.0 | 12.5-25 | 16.0 | 27.5-47.5 | 37.5 | . | . | 32-47.5 | 37.5 |
| 106337 | 87.7 | 93.6 | 27.5 | >60 | 4.5-24 | 15.0 | 12.5-17.5 | 15.0 | 22.5-27.5 | 23.3 | . | . | 15-25 | 22.5 |
| 106358 | 86.8 | 92.5 | 104.0 | >60 | 12.5-42.5 | 25.0 | 22.5-47.5 | 27.5 | . |  | . | . | . | . |
| 106361 | 87.1 | 92.0 | 75.0 | >60 | . | . | . | . | 8-27.5 | 17.5 | . | . | 2.5-75 | 32.5 |

CCL = curved carapace length, SCL = straight carapace length. IN = inter-nesting, m-IN = movement during IN, M = migration, m-F = movement during foraging, F = foraging; see Methods for how these behavioral modes were determined. “.” = not applicable or data not available. Dive durations were collected in bins, with the >60 min bin the largest bin set in tag settings.
