# Supplementary material for: Loggerhead sea turtle (Caretta caretta) diving changes with productivity, behavioral mode, and sea surface temperature
Source: PLoS One. 2019 Aug 7;14(8):e0220372. doi: 10.1371/journal.pone.0220372 (PMC6685635; doi:10.1371/journal.pone.0220372)
Supplement: S4 Table — For each dive behavior variable, the models are ordered by AICc from the smallest to the largest. (DOC) [file pone.0220372.s006.doc]

**S4 Table. Dive behavior model results for adult female loggerhead turtles.**

| **Frequency of all dives per day** | |  |  |  |
| --- | --- | --- | --- | --- |
| Variables | Pesudo-AICC | | Delta AICC | AICC weight |
| MODE, NPP, SST | 2231.68 | | 0.00 | 1.00 |
| MODE × SST, NPP | 2252.69 | | 21.01 | 0.00 |
| MODE, SST | 2261.00 | | 29.32 | 0.00 |
| MODE × SST | 2280.98 | | 49.30 | 0.00 |
| MODE × NPP, SST | 2333.83 | | 102.15 | 0.00 |
| MODE | 2476.41 | | 244.73 | 0.00 |
| MODE, NPP | 2472.11 | | 240.43 | 0.00 |
| SST × NPP, MODE | 2490.85 | | 259.17 | 0.00 |
| SST | 2314.79 | | 83.11 | 0.00 |
| SST, NPP | 2330.77 | | 99.09 | 0.00 |
| NPP | 2351.58 | | 119.90 | 0.00 |
| Null | 2511.90 | | 280.22 | 0.00 |
| SST × NPP | 2540.62 | | 308.94 | 0.00 |
| MODE × NPP | 2558.04 | | 326.36 | 0.00 |
| MODE × NPP × SST | 2589.34 | | 357.66 | 0.00 |
|  |  | |  |  |
| **Frequency of bottom dives per day** | | |  |  |
| Variables | Pseudo-AICC | | Delta AICC | AICC weight |
| MODE × SST, NPP | 2988.54 | | 0.00 | 0.89 |
| MODE, NPP, SST | 2992.63 | | 4.09 | 0.11 |
| SST × NPP, MODE | 3078.10 | | 89.56 | 0.00 |
| MODE, NPP | 3089.05 | | 100.51 | 0.00 |
| MODE × NPP, SST | 3122.62 | | 134.08 | 0.00 |
| SST, NPP | 3208.08 | | 219.54 | 0.00 |
| MODE × NPP × SST | 3221.82 | | 233.28 | 0.00 |
| MODE × NPP | 3244.26 | | 255.72 | 0.00 |
| MODE, SST | 3247.68 | | 259.14 | 0.00 |
| MODE × SST | 3253.14 | | 264.60 | 0.00 |
| SST | 3287.75 | | 299.21 | 0.00 |
| SST × NPP | 3340.51 | | 351.97 | 0.00 |
| Null | 3343.80 | | 355.26 | 0.00 |
| NPP | 3355.06 | | 366.52 | 0.00 |
| MODE | 3383.71 | | 395.17 | 0.00 |
|  |  | |  |  |
| **Frequency of long dives per day** | | |  |  |
| Variables | Pseudo-AICC | | Delta AICC | AICC weight |
| MODE, NPP, SST | 920.39 | | 0.00 | 0.98 |
| MODE, NPP | 928.70 | | 8.31 | 0.02 |
| MODE × SST, NPP | 936.21 | | 15.82 | 0.00 |
| MODE, SST | 956.77 | | 36.38 | 0.00 |
| SST × NPP, MODE | 938.32 | | 17.93 | 0.00 |
| MODE | 962.56 | | 42.17 | 0.00 |
| SST, NPP | 941.12 | | 20.73 | 0.00 |
| NPP | 947.03 | | 26.64 | 0.00 |
| SST × NPP | 955.25 | | 34.86 | 0.00 |
| MODE × SST | 972.49 | | 52.10 | 0.00 |
| MODE × NPP, SST | 983.86 | | 63.47 | 0.00 |
| SST | 988.83 | | 68.44 | 0.00 |
| MODE × NPP | 988.90 | | 68.51 | 0.00 |
| Null | 992.63 | | 72.24 | 0.00 |
| MODE × NPP × SST | 1010.12 | | 89.73 | 0.00 |
|  |  | |  |  |
| **TAD for surface dives per day** |  | |  |  |
| Variables | Pseudo-AICC | | Delta AICC | AICC weight |
| Null | 5841.40 | | 0.00 | 0.71 |
| MODE | 5843.20 | | 1.80 | 0.29 |
| SST | 5851.74 | | 10.34 | 0.00 |
| MODE, SST | 5853.96 | | 12.56 | 0.00 |
| NPP | 5864.93 | | 23.53 | 0.00 |
| MODE, NPP | 5866.86 | | 25.46 | 0.00 |
| MODE × SST | 5867.45 | | 26.05 | 0.00 |
| SST × NPP | 5871.54 | | 30.14 | 0.00 |
| SST × NPP, MODE | 5873.56 | | 32.16 | 0.00 |
| SST, NPP | 5874.59 | | 33.19 | 0.00 |
| MODE, NPP, SST | 5877.05 | | 35.65 | 0.00 |
| MODE × SST, NPP | 5890.56 | | 49.16 | 0.00 |
| MODE × NPP | 5899.41 | | 58.01 | 0.00 |
| MODE × NPP, SST | 5908.12 | | 66.72 | 0.00 |
| MODE × NPP × SST | 5919.94 | | 78.54 | 0.00 |

For each dive behavior variable, the models are ordered by AICc from the smallest to the largest.
