# Supplementary material for: Loggerhead sea turtle (Caretta caretta) diving changes with productivity, behavioral mode, and sea surface temperature
Source: PLoS One. 2019 Aug 7;14(8):e0220372. doi: 10.1371/journal.pone.0220372 (PMC6685635; doi:10.1371/journal.pone.0220372)
Supplement: S5 Table — The change is calculated for both the coldest SST and average SST values recorded (over a 1°C change) and the lowest and average values of NPP (over a 500 mg change). Dive metrics include the frequency of all dives, the frequency of bottom dives and the frequency of long dives. For SST, NPP was held constant at the average value (1,763 mg C square meter-1 day-1). For NPP plots, SST was held constant at the average value (29°C). In the table "mg C" is measured per square meter per day. (DOC) [file pone.0220372.s007.doc]

**S5 Table. The change in dive metric by behavioral mode in relation to sea surface temperature (SST) and net primary productivity (NPP).**

|  | **Change in dive metric** | | | |
| --- | --- | --- | --- | --- |
| **Dive metric** | **18 to 19 °C** | **29 to 30 °C** | **145 to 655 mg C** | **1760 to 2260 mg C** |
| **Frequency of all dives** |  |  |  |  |
| Migration | 1.22 | 2.33 | -1.26 | -1.14 |
| Inter-nesting | 1.40 | 2.67 | -1.44 | -1.31 |
| Foraging | 1.16 | 2.21 | -1.19 | -1.08 |
| **Frequency of bottom dives** |  |  |  |  |
| Inter-nesting | 0.40 | 0.56 | 1.26 | 1.67 |
| Foraging | 0.82 | 1.35 | 1.98 | 2.62 |
| **Frequency of long dives** |  |  |  |  |
| Migration | 0.26 | 0.30 | 0.15 | 0.16 |
| Inter-nesting | 0.25 | 0.29 | 0.15 | 0.15 |
| Foraging | 0.26 | 0.30 | 0.15 | 0.16 |

The change is calculated for both the coldest SST and average SST values recorded (over a 1 °C change) and the lowest and average values of NPP (over a 500 mg change). Dive metrics include the frequency of all dives, the frequency of bottom dives and the frequency of long dives. For SST, NPP was held constant at the average value (1,763 mg C square meter-1 day-1). For NPP plots, SST was held constant at the average value (29 ºC). In the table "mg C" is measured per square meter per day.
